# Optimisation of Potassium Chloride Nutrition for Proper Growth, Physiological Development and Bioactive Component Production in *Prunella vulgaris* L

**DOI:** 10.1371/journal.pone.0066259

**Published:** 2013-07-09

**Authors:** Yuhang Chen, Manman Yu, Zaibiao Zhu, Lixia Zhang, Qiaosheng Guo

**Affiliations:** 1 Institute of Chinese Medicinal Materials, Nanjing Agricultural University, Nanjing, P.R. China; 2 College of Pharmaceutical Sciences, Chengdu Medical College, Chengdu, P.R. China; 3 Structure Specificity of Small Molecules for Drug Research University Key Laboratory of Sichuan Province, Chengdu, P.R. China; United States Department of Agriculture, United States of America

## Abstract

*Prunella vulgaris* L. is an important medicinal plant with a variety of pharmacological activities, but limited information is available about its response to potassium chloride (KCl) supplementation. *P. vulgaris* seedlings were cultured in media with four different KCl levels (0, 1.00, 6.00 and 40.00 mM). Characteristics relating to the growth, foliar potassium, water and chlorophyll content, photosynthesis, transpiration, nitrogen metabolism, bioactive constituent concentrations and yield were determined after three months. The appropriate KCl concentration was 6.00 mM to result in the highest values for dry weight, shoot height, spica and root weight, spica length and number in *P. vulgaris*. The optimum KCl concentration resulted in a maximum net photosynthetic rate (Pn) that could be associated with the highest chlorophyll content and fully open stomata conductance. A supply of surplus KCl resulted in a higher concentration of foliar potassium and negatively correlated with the biomass. Plants that were treated with the appropriate KCl level showed a greater capacity for nitrate assimilation. The Pn was significantly and positively correlated with nitrate reductase (NR) and glutamine synthetase (GS) activities and was positively correlated with leaf-soluble protein and free amino acid (FAA) contents. Both KCl starvation (0 mM) and high KCl (40.00 mM) led to water loss through a high transpiration rate and low water absorption, respectively, and resulted in increased concentrations of ursolic acid (UA), oleanolic acid (OA) and flavonoids, with the exception of rosmarinic acid (RA). Moreover, the optimum concentration of KCl significantly increased the yields of RA, UA, OA and flavonoids. Our findings suggested that significantly higher plant biomass; chlorophyll content; Pn; stronger nitrogen anabolism; lower RA, UA, OA and flavonoid accumulation; and greater RA, UA, OA and flavonoid yields in *P. vulgaris* could be expected in the presence of the appropriate KCl concentration (6.00 mM).

## Introduction

*Prunella vulgaris* L., also known as “self-heal”, is a perennial herb and constitutes an important species of the genus *Prunella* from the Labiatae. In ancient Chinese, it is known as ‘Xiaku-cao’, which means ‘plant that will wither and die after the summer period' [1]. This is a thermophilic and hygrophilous species that is native to eastern Asia, which is most heavily distributed in the middle, south-eastern and south-western provinces and regions of China [2]. It can often be found in ravines, stream sides, forests, wet meadows, slopes, thickets, field margins, trail sides, roadsides or ditch banks in the provinces of southern China [3], [4].

*P. vulgaris* has been identified as one of the most promising potential medicinal and edible wild plant resources [5]. The fresh leaves of young plants are popular for use in a wild vegetable recipe in southeast China [4], whereas its mature plants are commonly used as a traditional medicinal herb in some Asian and Europe countries, such as China [6], Korea [7], Japan [8], Turkey [9], the Czech Republic [10], Poland [11] and Germany [12]. *P. vulgaris* is rich in phenolic acids [13]. Rosmarinic acid (RA), which is the major phenolic component of *P. vulgaris*, exhibits a wide spectrum of biological activities [14], including the suppression of lipoperoxidation [15], superoxide radical scavenging [16] and antioxidant [17] and anti-inflammatory [18] effects. The Chinese Pharmacopoeia of 2010 considers RA to be the only criterion for Spica Prunellae (*P. vulgaris* fruiting spikes) quality control. In addition, triterpenes are the dominant compounds in *P. vulgaris* [19]. Of the triterpenes, ursolic acid (UA) and oleanolic acid (OA) are most prevalent in *P. vulgaris* and exhibit many bioactivities, including hepatoprotection and anti-hyperglycemia, antifungal, antitumour and anti-inflammatory effects [20], [21]. In addition, the plant's total flavonoids have been reported to exhibit a wide range of biological effects, including antioxidant, antibacterial, antiviral, anti-inflammatory, anti-allergic and vasodilator actions [22], [23].

In response to these discoveries, the demand for *P. vulgaris* has been increasing in the last several years. However, the wild resources in China cannot meet such a huge demand. In recent years, farmers have been trying to increase production by artificial cultivation [1]. Because *P. vulgaris* is different from field crop plants such as cotton, wheat, maize and rice [24], [25], [26], [27], [28], only limited information is available about its response to different fertilisation regimes. Therefore, it is necessary to establish an efficient fertilisation system for commercial cultivation. However, the effects of different potassium chloride (KCl) supplement levels on the plant's growth, physiology and secondary metabolites are largely unknown. Therefore, the main objective of the present study was to discern the optimal KCl supplement level for *P. vulgaris* growth by investigating the effects of different KCl levels on the growth, foliar K concentrations, water and chlorophyll concentrations, photosynthesis, transpiration, nitrogen metabolism, RA, UA, OA, flavonoid concentrations and yield.

## Materials and Methods

### Plant materials and growth conditions

A hydroponic experiment was conducted in the greenhouse at the Institute of Chinese Medicinal Materials at Nanjing Agricultural University, Nanjing, P.R. China. Seeds from *P. vulgaris* were collected from Queshan County, Henan Province in June of 2009. Hydroponic culture experiments were conducted in a growth chamber with a 12 h photoperiod at 27/20°C (day/night) temperatures, 60–70% relative humidity, and 1000 µmol m^−2^ s^−1^ of photosynthetically active radiation. Seeds were surface-sterilised with 9% H_2_O_2_ for 30 min and germinated in propagation pots during October of 2009. On March 28th 2010, after approximately 10 days of cultivation in the hydroponic solution, seedlings of uniform sizes were selected and cultured on MS [29] media with four different potassium levels (0, 1.00, 6.00 and 40.00 mM) from KCl. The 0 mM KCl treatment was the negative control. Each pot contained eight seedlings. A completely randomized design was used with five replications (pots). All solutions were completely renewed every 7 days, and deionised water was added daily to replace water lost by evapotranspiration. The solution pH was maintained at 6.5 by addition of concentrated NaOH solution, and the solution was continuously aerated with an air pump to provide O_2_ and achieve nutrient homogeneity. Plants were harvested after three months to determine their growth characteristics, foliar K concentrations, water and chlorophyll contents, photosynthesis and transpiration parameters, nitrogen metabolism, bioactive constituent concentrations and yields.

### Growth parameters and water contents

Plant water content and growth parameters including the dry weight, shoot height, spica and root weight, spica length and number were measured. The shoot height and spica length were determined with a vernier calliper. The shoot height indicates the value between the top of the plantlet and the stem base. The length of the main inflorescence from each individual plantlet represents the spica length.

### K concentrations

The second fully expanded leaves were used to determine the K concentration. Samples were dried as follows: first, the fresh leaves were fixed quickly at 105°C for 30 min, and then they were oven-dried at 70°C for 48 h. The dried material was milled to pass a 0.5 mm sieve. The powder (0.10 g) was digested with nitric acid and H_2_O_2_ using a Milestone Ethos Touch microwave digestion system (Ethos touch control, Milestone Inc., Italy). The extraction solution was used to determine the K concentration with an inductively coupled plasma atomic emission spectrometer (ICP-AES, Optima 2100 DV, Perkin Elmer, USA). The foliar K content was calculated in terms of mg g^−1^ of dry weight (DW).

### Chlorophyll content

Pigments were extracted according to previous report [30]. Chlorophyll concentrations were estimated from the second fully expanded leaves from the top of the individual plants. One gram of fresh leaf tissue was homogenised with 80% (v/v) acetone (10 ml) and centrifuged. The pigment concentration was determined in mg g^−1^ using a spectrophotometer.

### Photosynthesis and transpiration parameters

The measurements were carried out from 09:00 to 11:30 a.m. with the second fully expanded leaves for the determination of net photosynthetic rate (Pn), stomatal conductance (Cond), transpiration rate (Tr) and intercellular CO_2_ concentration (C_i_). These parameters were measured with a portable photosynthesis system (LI-6400, Li-Cor, Lincoln, NE, USA) with the air temperature, relative humidity, CO_2_ concentration and light intensity inside the leaf chamber maintained at 25°C, 60%, 500 µmol CO_2_ mol^−1^, and 1000 µmol m^−2^ s^−1^, respectively.

### Nitrogen assimilation and recycling

Fresh samples from the most fully expanded leaves were frozen in liquid N for 1 min and then stored at −80°C for soluble protein and free amino acid (FAA) content determination, as well as the key enzyme activity assay. Approximately 1 g of leaves was homogenised with 10 ml of 50 mM sodium phosphate, pH 7.8 that contained 2 mM EDTA and 80 mM L-ascorbic acid. After centrifugation at 15,000 × G for 20 min, the supernatants were used to determine the soluble protein, FAA content, and key enzymes activities [31], [32].

The nitrate reductase (NR) activity was determined as described in previous publications [33]. In brief, the reaction medium (with a total volume 1 ml) consisted of 50 mM sodium phosphate, pH 7.8, 10 µM FAD, 1 mM DTT, 5 mM KNO_3_, and 20 mM EDTA. The reaction was initiated by adding 200 µl of extract and terminated after 5 min by adding 125 µl of zinc acetate solution (0.5 M). Nitrite formation was measured colorimetrically by adding 750 µl of 1% sulphanilamide in 3 M HCl and 750 µl of 0.02% N-naphthyl-ethylenediamine hydrochloride, and the absorption was determined at 546 nm.

Glutamine synthetase (GS) activity was determined by means of the synthetase reaction [34]. The volume of the reaction mixture was 2.2 ml, including 0.5 ml of enzyme extract. Hydroxamate formation was measured in the assay mixture after 15 min at 30°C. The absorbance was measured at 540 nm.

### RA, UA, OA and total flavonoid metabolism

The method of extraction and quantification for total flavonoid content followed the previous method [35]. An amount of ground spica samples (0.10 g) was extracted with 35% ethanol (10 ml) on an orbital shaker for 120 min at 50°C. A sample (1 ml) was mixed with 5% NaNO_3_ (0.3 ml) in a test tube that was covered with aluminium foil and left for 6 min, then 10% AlCl_3_ (0.3 ml) was added, followed by the addition of 4% NaOH (4 ml). The absorbance was later measured at 510 nm with a spectrophotometer, with rutin as a standard.

Samples for HPLC determination were prepared by modifying the previous research methods [5]. Powdered spica samples (0.10 g) were mixed with 20 mL of 75% methyl alcohol for 30 min and then extracted over the course of a 30 min ultrasonic treatment at 20°C and centrifuged at 10,000 rpm for 10 min. The upper solution was filtered through a 0.45 µm organic membrane filter before being injected into the HPLC system.

The RA, UA and OA contents were determined using the HPLC system, namely an LC-20AT Liquid Chromatograph (Shimadzu, Kyoto, Japan). The methods for determining these three bioactive components have been previously described [5]. The total flavonoid, RA, UA and OA yields were calculated by multiplying the total flavonoid content, RA, UA and OA in the spicas by the dry weight of the spica.

### Statistical analysis

The resulting values were presented as the means ± standard error (SE) from these independent treatments. These data were subjected to an analysis of variance, correlation and Duncan's multiple range tests (*P*<0.05) with SPSS 18.0 for Windows.

## Results

### Growth parameters

The *P. vulgaris* six growth parameters of interest were the dry weight, shoot height, spica and root weight, spica length and number, all of which exhibited significant differences with each treatment (Table 1). The treatment with 6.00 mM KCl presented the highest values for these six growth parameters, whereas potassium starvation (0 mM) severely inhibited the growth of *P. vulgaris*. Additionally, a high KCl concentration (40.00 mM) also caused severe reduction in these parameters. The minimum individual plant dry weight, spica and root weight, shortest shoot height and lowest spica number were found in the potassium starvation treatment (0 mM), whereas the shortest spica length was also found in the treatment with the highest KCl level (40.00 mM).

**Table 1:** Effects of different potassium chloride supplies on foliar K, water and chlorophyll contents and growth parameters of *P. vulgaris*.

	K0	K1	K2	K3
Dry weight (g plant^−1^)	1.49±0.02 d	5.41±0.21 b	6.13±0.14 a	1.95±0.03 c
Shoot height (cm)	13.16±0.30 c	19.96±2.67 b	23.94±1.45 a	14.89±0.42 c
Spica weight (g plant^−1^)	0.34±0.03 c	2.16±0.34 b	2.96±0.16 a	0.48±0.03 c
Spica length (cm)	2.21±0.42 c	3.25±0.32 b	3.74±0.27 a	1.86±0.27 d
Spica number	5.70±1.16 c	14.80±3.36 b	18.10±5.11 a	7.78±2.28 c
Root weight (g plant^−1^)	0.29±0.02 c	0.43±0.03 b	0.58±0.02 a	0.31±0.02 c
Foliar K (mg g^−1^ DW)	19.21±0.61 d	25.26±0.63 c	44.97±0.21 b	66.21±0.89 a
Water content (g 100 g^−1^)	61.64±0.05 c	79.29±0.03 a	73.81±0.06 b	73.07± 0.02 b
Chlorophyll content (mg g^−1^ FW)	1.25±0.01 d	1.45±0.01 b	1.66±0.07 a	1.38±0.04 c

Note: Each value represents the mean ± SE (n = 10). The values that are followed by the different letter in the same lines are significantly different according to Duncan's multiple range test (*P*<0.05). K0, K1, K2 and K3 indicate 0, 1.00, 6.00 and 40.00 mM KCl, respectively.

### Leaf K, water and chlorophyll contents

Foliar K contents were significantly affected by the supplemental levels of KCl (Table 1). The maximum leaf K concentrations were recorded in the 40.00 mM KCl treatment, and the higher KCl supplementation led to increased potassium absorption in comparison to the 0 mM KCl treatment. However, the maximum potassium absorption did not have the maximum dry matter quantity (Table 1).

Plant water contents were also significantly different between treatments (Table****1). The maximum water content was observed in the 1.00 mM KCl treatment, whereas both the no potassium and high KCl (>1.00 mM) treatments resulted in plant water loss.

The maximum chlorophyll content was recorded for 6.00 mM KCl (Table****1), whereas the lowest chlorophyll was observed for the no potassium treatment and experienced a significant increase with the higher KCl levels (>1.00 mM). Finally, the chlorophyll content dropped to the next minimum level at 40.00 mM.

### Photosynthesis and transpiration parameters

The net photosynthetic rate was at a maximum for the 6.00 mM KCl treatment, and it dropped dramatically for all the other treatments (Table****2). The plants in this treatment had the lowest intercellular CO_2_ concentration (Table****2), which represented the higher carbon assimilation and could have directly contributed to the dry matter production. Meanwhile, the maximum net photosynthetic rate was positive and significantly correlated with fully open stomata conductance, and the Pearson correlation coefficient was 0.99. There was a positive significant correlation between the net photosynthetic rates and chlorophyll content, and the Pearson correlation coefficient was 0.98 (Table****2).

**Table 2:** Effects of different potassium chloride supplies on net photosynthetic rate (Pn), intercellular CO_2_ concentration (C_i_), transpiration rate (Tr) and stomatal conductance (Cond) in the leaves of *P. vulgaris*.

	K0	K1	K2	K3
Pn (µmol CO_2_ m^−2^ s^−1^)	7.67±0.39 d	10.53±0.41 b	12.80±0.61 a	8.99±0.26 c
C_i_ (µmol CO_2_ mol^−1^)	233.51±2.54 d	259.83±5.69 a	252.48±1.56 b	238.09±2.21 c
Tr (mmol H_2_O m^−2^ s^−1^)	4.35±0.20 a	2.89±0.88 bc	2.39±0.19 c	3.69±0.31 b
Cond (mol H_2_O m^−2^ s^−1^)	0.10±0.01 c	0.17±0.05 ab	0.21±0.01 a	0.13±0.02 bc

Note: Each value is presented as the mean ± SE (n = 6). Values that are followed by a different letter in the same line are significantly different according to Duncan's multiple range test (*P*<0.05). K0, K1, K2 and K3 indicate 0, 1.00, 6.00 and 40.00 mM KCl, respectively.

Transpiration rates were significantly different between the potassium starvation state and the other three KCl treatments (Table****2). However, a maximum stomatal conductance was observed for 6.00 mM KCl (Table****2). This finding suggests that the appropriate KCl concentration strongly stimulated stomatal opening and then closed them, as was observed in the other three treatments.

### Nitrogen assimilation and recycling

The NR and GS activities were significantly affected by KCl treatments (Table****3). The maximum NR and GS activities were recorded from the 6.00 mM KCl treatment. However, the NR and GS activities were low in both the absence of potassium as well as the high KCl treatment (40.00 mM). The net photosynthetic rates were significantly and positively correlated with NR and GS activities, and the Pearson correlation coefficient were 0.97, 0.94, respectively.

**Table 3:** Effects of different potassium chloride supplies on nitrate reductase (NR), glutamine synthetase (GS), and the concentrations of soluble protein and free amino acids (FAA) in the leaves of *P. vulgaris*.

	K0	K1	K2	K3
NR (µg g^−1^ FW h^−1^)	5.65±0.31 c	7.33±0.33 b	9.06±0.42 a	6.97±0.74 b
GS (Unit g^−1^ FW h^−1^)	0.10±0.01 b	0.13±0.01 a	0.14±0.02 a	0.12±0.01 ab
Soluble protein (mg g^−1^ FW)	8.57±0.18 c	9.03±0.46 c	12.77±0.23 a	11.31±0.35 b
FAA (mg 100 g^−1^ FW)	9.46±0.58 c	11.99±0.81 b	13.56±0.48 a	13.05±0.95 ab

Note: Each value is the mean ± SE (n = 6). Values that are followed by a different letter in the same line are significantly different according to Duncan's multiple range test (*P*<0.05). K0, K1, K2 and K3 indicate 0, 1.00, 6.00 and 40.00 mM KCl, respectively.

The concentration of soluble protein and FAA were significantly affected by the KCl concentrations (Table****3). Both soluble protein and FAA contents were at their maximum in the 6.0 M KCl treatments, whereas they decreased sharply in all other treatments (Table****3). This finding suggests that there was a greater capacity for nitrogen assimilation and protein degradation under KCl-sufficient (6.00 mM) solutions, as indicated by the higher NR activity, higher soluble protein and higher FAA content (Table****3). The net photosynthetic rates were positively correlated with soluble protein and FAA contents, and the Pearson correlation coefficients were 0.71 and 0.76, respectively.

### RA, UA, OA and total flavonoid metabolism

The RA, UA, OA and total flavonoids were significantly affected by the different KCl treatments (Table****4). The maximum RA and total flavonoid contents were recorded from the 0 mM KCl treatment. The RA and total flavonoid contents showed a continuous reduction in the other three treatments in association with their KCl levels. Both the UA and OA activities were low for the 6.00 mM KCl treatment, and both the absence of potassium as well as high KCl stimulated their accumulation (Table****4).

**Table 4:** Effects of different potassium chloride concentrations on the contents of rosmarinic acid (RA), ursolic acid (UA), oleanolic acid (OA) and total flavonoids in the spicas of *P. vulgaris*.

	K0	K1	K2	K3
RA (%)	0.541±0.012 a	0.254±0.005 c	0.384±0.017 b	0.146±0.013 d
UA (%)	0.141±0.004 a	0.139±0.010 a	0.104±0.008 b	0.137±0.003 a
OA (%)	0.035±0.003 a	0.022±0.009 b	0.018±0.003 c	0.037±0.004 a
Total flavonoids (%)	6.862±0.038 a	4.344±0.010 d	5.082±0.033 c	6.502±0.027 b

Note: Each value represents the mean ± SE (n = 3). Values that are followed by a different letter in the same line are significantly different according to Duncan's multiple range test (*P*<0.05). K0, K1, K2 and K3 indicate 0, 1.00, 6.00 and 40.00 mM KCl, respectively.

### RA, UA, OA and total flavonoid yields

The RA, UA, OA and total flavonoids are the four major bioactive constituents in *P. vulgaris*. The levels of these constituents are important criteria for determining the quality of *P. vulgaris*. Therefore, the RA, UA, OA and total flavonoids were determined by multiplying the spica contents by the spica dry weights (Table****5). The RA, UA, OA and total flavonoid yields were significantly affected by the KCl treatments (Table****5). The maximum RA, UA, OA and total flavonoid yields were recorded for the 6.00 mM KCl treatment, whereas they were reduced sharply in all other treatments (Table****5).

**Table 5:** Effects of different potassium chloride supplies on yields of rosmarinic acid (RA), ursolic acid (UA), oleanolic acid (OA) and total flavonoids in the spicas of *P. vulgaris*.

	K0	K1	K2	K3
RA (g plant^−1^)	0.183±0.004 c	0.548±0.009 b	1.138±0.050 a	0.070±0.006 d
UA (g plant^−1^)	0.048±0.001 c	0.300±0.022 a	0.307±0.023 a	0.066±0.001 b
OA (g plant^−1^)	0.012±0.001 b	0.048±0.002 a	0.052±0.008 a	0.017±0.002 b
Total flavonoids (g plant^−1^)	2.342±0.013 d	9.395±0.022 b	14.943±0.097 a	3.109±0.013 c

Note: Each value is equal to the mean ± SE (n = 3). Values that are followed by a different letter in the same line are significantly different according to Duncan's multiple range test (*P*<0.05). K0, K1, K2 and K3 indicate 0, 1.00, 6.00 and 40.00 mM KCl, respectively.

## Discussion

The main aim of this experiment was to investigate the effects on the *P. vulgaris* growth performance, physiology development and bioactive component production of KCl as sole source of potassium. The results of this study coincide with our earlier finding that KCl in a pot experiment enhances spicas appearance and improves spicas quality of *P. vulgaris* [1], [36]. Our leaf analyses also showed significantly higher Cl accumulation in KCl-treated plants, the highest Cl levels being recorded in the 40.00 mM KCl-treated plants (date not shown), however, Cl concentrations remained below the toxic level for *P. vulgaris*, and no Cl toxicity was detected in either treatment. Cl increases the incidence of gold speck injury [37], but this phenomenon was not observed here.

An optimum concentration of potassium is essential for *P. vulgaris* growth. Many studies have demonstrated an obvious relationship between plant stature and yield to the proper supply of potassium [38], [39], [40], [41], [42]. In the present study, *P. vulgaris* plants acquired the highest values for their dry weight, shoot height, spica and root weight, spica length and number with 6.00 mM of KCl, and as such this concentration represented the most favourable treatment for growth and development. Potassium starvation as well as its surplus caused a severe reduction in plant growth and development. The length and surface area of the roots were affected by the range of nutrient absorption, which had direct negative consequences on plant productivity [24], [43], [44]. Therefore, decreases in the dry weigh, shoot height, spica weight, spica length and number of *P. vulgaris* plants were partly due to changes in the root length and number. Moreover, although the supply of surplus KCl resulted in higher concentrations of foliar potassium when compared to 6.00 mM KCl, the higher leaf K did not increase the biomass. This finding might lend support to a hypothesis that the plant most likely needs a critical cytoplasmic concentration of K within a certain range [45].

Photosynthesis was significantly affected by potassium concentrations, which ultimately determined overall yield. It is well known that photosynthesis is related to carbon assimilation and dry matter production [46], [47], [48] and that the chlorophyll content, chloroplast ultrastructure and stomatal conductance are the major factors in determining the photosynthesis rate [49], [50]. Meanwhile, the treatment with the optimum concentration of KCl in the present study did experience an increase in stomatal conductance, and wrinkled leaves were observed in the treatments with potassium starvation and high KCl, which might be due to water loss and therefore represent poor chloroplast ultrastructure (which was not measured in this study). Many studies have shown that the appropriate potassium concentration can enhance chlorophyll content and photophosphorylase activity. The right amount of potassium can maintain the chloroplast inner membrane and the proton gradient of thylakoid membranes, which promote photosynthetic phosphorylation [44], [51], [52], [53]. Therefore, the maximum net photosynthetic rate from the 6.00 mM KCl treatment may be mainly associated with the high chlorophyll content, stable chloroplast ultrastructure and fully open stomata conductance.

The appropriate KCl concentration could maintain a critical water concentration in *P. vulgaris*. It is well known that potassium plays an important role in controlling stomatal aperture because the concentration gradient of potassium between the inside and outside of stomatal guard cells affects the solute potential [54], [55]. In the present study the treatment with the optimal KCl concentration had a lower transpiration rate than the treatment with potassium starvation, resulting in higher water content, which is important for cell functions. Meanwhile, high KCl resulted in lower water content in comparison to the 1.00 mM KCl treatment. This finding suggested that high KCl reduced water absorption.

Because NR activity is positively associated with photosynthesis and GS plays a positive role in the link between carbon and nitrogen metabolism [56], potassium can increase both NR expression and its activity [33] and GS activity [57]. In the present study, KCl markedly increased the activities of NR and GS, particularly at the appropriate KCl concentration, which occurred in parallel with a rise in photosynthesis. Meanwhile, our results showed that the appropriate KCl concentration did rise significantly in soluble protein content, so K-sufficient solutions lead to stronger photosynthesis by increasing the proportion of soluble protein to total leaf N, which favourably affects Rubisco protein and activity [58]. Furthermore, we noted that the appropriate KCl concentration led to an increase in the accumulation of FAA (Table****3). Moreover, because net photosynthetic rates were positively correlated with the leaf-soluble protein and FAA contents, the increase in the latter under the appropriate KCl concentration may be responsible for the increase in photosynthetic capacity.

Potassium starvation and high potassium conditions cause oxidative stress due to the formation of reactive oxygen species (ROS; e.g., O_2_^•−^ and H_2_O_2_) [43], [59], [60], [61]. To protect themselves against the injurious effects of ROS, plants have evolved complex antioxidant system which includes enzymatic antioxidants and non-enzymatic antioxidants [62]. The accumulation of secondary metabolites is known to be a defence mechanism that can help plants to respond and adapt to oxidative stress by altering cellular metabolism to face various challenges [63]. A large body of evidence indicates that extracts of *P. vulgaris* possess antioxidant activity. Previous studies have observed that 30% aqueous ethanol extract of *P. vulgaris* with RA is able to suppress lipopolysaccharide (LPS)-induced oxidative damage [64]. Similarly, previous publications found that the highest extraction yield of flavonoids in *P. vulgaris* spicas were detected in a 41% aqueous ethanol extract, in which the best inhibitory effect of lipid peroxidation, the highest DPPH and hydroxyl free radical scavenging activities were observed [65]. Additionally, a pharmacological experiment showed that UA and OA are triterpenes with similar chemical structures, possessing scavenging activity against reactive oxygen species and inhibiting peroxidation in rat liver homogenate [66], [67]. Furthermore, terpenoids have been shown to possess antioxidant properties in different situations, particularly against lipid peroxidation, as a result of their high lipophilicity [68]. Based on this information, the free radical scavenging and membrane-stabilising capacity of RA, UA, OA and flavonoids cause oxidative stress when exposed to potassium starvation and high KCl, so an optimal concentration of KCl is needed for their physiological and pharmacological function.

As predicted by the Growth/Differentiation Balance Hypothesis (GDBH), rapidly growing plants have a low secondary metabolite concentration as a result of a resource-based trade-off between primary and secondary metabolic pathways. Because environmental stress reduces plant growth, the carbon that is fixed during photosynthesis can be used for the formation of secondary compounds [69]. Potassium deficiency limitation slows growth more than carbon assimilation, which can result in the accumulation of carbohydrates in source leaves. This response may increase the substrate that is available for secondary metabolism. However, when resource limitation is severe enough to depress carbon assimilation, secondary metabolism is predicted to fall because of the energy and substrate concentration that is needed for biosynthesis [70]. Coinciding with this prediction, the RA, UA, OA and flavonoid contents increased with potassium starvation, whereas RA decreased under the highest KCl condition (40.00 mM).

In the present study, potassium starvation and high KCl (40.00 mM) significantly reduced spica dry weight per plant at the harvest period, but increased the bioactive constituents of *P. vulgaris*, other than the RA content. The appropriate potassium concentration could maintain a critical concentration of RA, UA, OA and flavonoids and promote the highest amounts that were found in *P. vulgaris* spicas. Consequently, the maximum RA, UA, OA and flavonoid yields were measured from the 6.00 mM KCl treatment, whereas they were reduced sharply in all other treatments.

Despite a few decades of research, the enzymes, genes, and biochemical pathways that are involved in RA, UA, OA and flavonoid biosynthesis remain largely uncharacterised. The results of the current investigation suggest the high potential for using suitable potassium concentrations as a strategy for increasing the yield of RA, UA, OA and flavonoids. To meet the strong market demand for these bioactive products, regulating the biosynthesis of RA, UA, OA and flavonoids in terms of their possible function in hydroponic cultural systems may be a feasible strategy to bypass the low product yield bottleneck.

In summary, our results have demonstrated that a suitable KCl concentration (6.00 mM) for *P vulgaris* can enhance the net photosynthetic rate, chlorophyll content and nitrogen assimilation of the plant and cause a decrease in the levels of RA, UA, OA and flavonoids, but it leads to an increase in the RA, UA, OA and flavonoid yields. This finding suggests that the application of the appropriate KCl concentration (6.00 mM) to the nutrient solution is important for improving the biomass production and bioactive constituent yield of *P vulgaris*. This study realizes the need of further study about the interaction effect of K and Cl in *P. vulgaris* in a hydroponic culture system. The elaborate physiological and biochemical mechanism in which RA, UA, OA and flavonoids play a crucial role under potassium or other nutrient solution with varying nitrogen and phosphorus concentrations remains unclear and needs to be addressed in the future. In addition, to increase the yields of RA, UA, OA and flavonoids from *P. vulgaris*, a production protocol consisting of the application of potassium fertiliser at a suitable amount and at the most relevant phenological stages should be investigated.
